# Supplementary material for: Patterns of Intron Gain and Loss in Fungi
Source: PLoS Biol. 2004 Nov 30;2(12):e422. doi: 10.1371/journal.pbio.0020422 (PMC532390; doi:10.1371/journal.pbio.0020422)
Supplement: Table S1 — Also available at http://genes.mit.edu/NielsenEtAl/. (4.3 MB ZIP). [file pbio.0020422.st001.zip › NielsenEtAl/html/962.html]

AN8787.1.NCU00134.1.MG10127.1.FG06659.1


```
 CLUSTAL W (1.82) Multiple Sequence Alignments - Introns Inserted


Sequence 1: NCU00134.1	1018 aa
Sequence 2: MG10127.1	1032 aa
Sequence 3: FG06659.1	1022 aa
Sequence 4: AN8787.1	1031 aa
Alignment Length: 1045 aa
Number Identitical Residues: 397 aa
Alignment Score (without introns) 22312


MG10127.1 	MDAQ0IENAIQIAWDPQSDPSLKSQAFEFLQQLRADPSAWHICSTLFTKSPRSADVVRLV
NCU00134.1	MDAQ0FEKAIEIAFDPRSSHALKSQALEFLNQVRTDVQAWRICAALFTRSPRASDIVRHV
FG06659.1 	MEAQ0IENAVGILSNPTSDRSINAQAFEYLNQLRTDPSGWQACTTLFARTPRTLEVVRLV
AN8787.1  	MEEQ0VANAIEIAGNPTSDSTLKAQAFDYLNQLRTDPSGWQVCLSLFTKDPPQSHFIRHV
          	*: * . :*: *  :* *. ::::**:::*:*:*:* ..*: * :**:: *   ..:* *

MG10127.1 	SLEIVNLAVQVQQLDAASLAFLKDSLLDYVRRTYGPGAQDEPDVPSLQNKLTQTLTYLFA
NCU00134.1	SLEMVNNAVHSQGLDAPDLAFVKNSLLDYITRTYGPNAQDQADPANVQNKLTQTLTYLFV
FG06659.1 	CLEVVNYAVHTQGLDQASLGFLKDTLLQYCRQTYGPNAQQEADPAHLQNKLTQTLTYLFV
AN8787.1  	SLEVVNSAAQAGLIDLRSLGFVRDRLLAYLRQVYGREGSN-PDPPNIQNKIAQTITFLFS
          	.**:** *.:   :*  .*.*::: ** *  :.**  ..: .* . :***::**:*:** 

MG10127.1 	RLYKSGWETFLSDFLALTASSEGNPRQRDNVRGVTLYLRILGSVHDEIADNMLARQGNEG
NCU00134.1	ALYGEGWETFFDDFLALTSSQNGASR--DNLSGVMLYLRILSSVHDEIADLMISRQGNES
FG06659.1 	FLYQNGWESFLDDHLELT----GLSTNTDNVQGVIFYLRILGSIHDEIADMLLSRQNNEA
AN8787.1  	ALYGNGWESFFDDLLSLTHKS-PSSTTRDNPLGIIFYLRVINSIHDEIGDVLVSRSRAEQ
          	 ** .***:*:.* * ** ..   .   **  *: :***::.*:****.* :::*.  * 

MG10127.1 	KRNAELKDAIRERDMRMVVESWQDLLSQYTGRDDAILEHTLKVMAKWVSWIDISLVITQD
NCU00134.1	KRNNDLKDLIRERHMQKIAMSWQDILAQWTNKHDGVVELTLKVIGKWVSWIDISLVVSQD
FG06659.1 	KRNTELKDQLRAQDMHKVCESWKQLLTRYS-TNDTVVELVLKVIGKWVSWMDIALVISQD
AN8787.1  	ERANALKDLIRVRDMQKIASSWQEILSQWMDGDDLIVEMSLKAVGSWVSWIDIGLVVNQT
          	:*   *** :* :.*: :  **:::*::: . .* ::*  **.:..****:**.**:.* 

MG10127.1 	MLNLLFPLIGCVN---PQGGADMVRDAAIEAFTEIAGKKMKPVDKTELISFLNLRQIISE
NCU00134.1	MQNLILPLVGRVNN--TSNNIDTVRDTAIDTLTEIVAKKMGPSHKMELISFLNLGGIITE
FG06659.1 	MLGLLLPVVGRPS----TSGEDKVRDTAIDTLNEICGKKMRSSDKMEMISFINLQEIVEQ
AN8787.1  	MLDLLFQQLGRAQKAELREGEDKVRDAAVDVFTEIIGKKMKAEDKIDMIAFLNLDNVVGQ
          	* .*::  :*  ..:.   . * ***:*::.:.** .*** . .* ::*:*:**  :: :

MG10127.1 	LVASPPLSQFRGTHRYDSDLAESVAKLVNVVVTDVVKVLEDGSVGEESRAKAGQHLQDFL
NCU00134.1	LLASQGLHEFKGTSRYDNDLAEVVAKLLNTIMTDVVRVLEDNKVDAETRAKAERHLQDFL
FG06659.1 	LVASPPLSEYKGTSRYDTDLAEAVAKLVNTVLSDIVRALEDSQISDDTRARANQHLHGFL
AN8787.1  	ISSSPPLYANRFTSKYDTDLAETVAKLVNTTVTDIVRALEQETVSAQCKEKANGLLQVFL
          	: :*  *   : * :**.**** ****:*. ::*:*:.**:  :. : : :*   *: **

MG10127.1 	PLLLRLFSDEYDEVCSTVIPSLTDILTFLRKVP----ELPDSYREMLRPIMDAIVAKMRF
NCU00134.1	PALLRLFSDEFDEVCSTVIPSLTDLLTFLRRVG----TLPDSYSQMLRPILSAIVAKMRY
FG06659.1 	PFLLRFFSDEYDEVCSSVIPALTDLLTFLRKLT----VVNQDYGGMLAPILDAIIRKMRY
AN8787.1  	PHILRYFSDEYDEVCSTVIPCVSDLLSYLRKMAKSNPSIASQHSSILLPILKAIIQKMRY
          	* :** ****:*****:***.::*:*::**:: .:.. : ..:  :* **:.**: ***:

MG10127.1 	DETSHWFEGEEAEEEADFLELRKRLQNLQKSVAAVDENLFIDVMSNLVATTLQNLDERGA
NCU00134.1	DETSSWGTEDGESDEAEFQELRKRLQILQKSVAAVDQTLYIEFLSNLVGNMFATLEQQGP
FG06659.1 	DETTTWGDEDEVTDEAEFQDLRRKLQNLQKSIAAIDQPLYMDMLSNLVATTFSTLDQQGS
AN8787.1  	DETASWGDDDDQTDEAEFQELRKRLGTLQQIVAAVDERLYMEAVSEVVATTFENMRQSGA
          	***: *   :   :**:* :**::*  **: :**:*: *::: :*::*.. : .: : *.

MG10127.1 	QMDWRDIDLALHELLQFGELALPNQGLAAKSQPSSNAAERLNVIMRKLVESS1IADFPHP
NCU00134.1	QMDWRDLDLALHEIYLFGELALPNAGLAHKSEPNVVATERLAVMMSKMVES-~-------
FG06659.1 	RMDWRDLDLALYEMYLFGELALPNQGLGTKNQPSTEASERLVVMMQKMVESG1IANFSHP
AN8787.1  	QLDWRDLDLALHEMYLFGDSATKSGSLYNKGQPSGPSAERLVEMMLRMVESD~IRSFTHP
          	::****:****:*:  **: *  . .*  *.:*.  ::***  :* ::***.   . . .

MG10127.1 	A~VLLQYMEVCVRYCVFFETNH-SYIPRVLENFVRLVHYDLTRFRVRSWYLFHRFVKQLR
NCU00134.1	A1ILLQYMEICVRYHAFFESHH-QYIAPVLENFVHLIHHEHPRVRTRSWYLFLRFVKQLR
FG06659.1 	A~ILLQYMEICVRYCVVFENHLSQYIPQVLENFVRLVHHDHVRIKTRSWYLFHRFIKQLR
AN8787.1  	A~TQLQYMEICVRYSSFFHTHT-HLIPGVLESFLQLAHHPMKKVKTRAWYLFQRLVKQLR
          	*   *****:****  .*..:    *. ***.*::* *:   :.:.*:**** *::****

MG10127.1 	AQVGNVAETIIQSIADLLPIKAEVSAEDGSEDDMSSDQTDNSADAIFNSQLYLFEAIGYI
NCU00134.1	AQVGNVAKTVIQSISDLLPIKAEVPS-TEAEDDMSSDESDHSADAIFNGQLYLFEAIGCI
FG06659.1 	AQVGNVAETVIQSIGDLLPIKAEVPG-DDADDDMSSDESDHSADALFTSQLYLFEAIGCI
AN8787.1  	AYIDNVAQTVVEALGDLLVIQAELPSESSDGDEMSSEDHEGSTDAVFNSQLYLFEAVGII
          	* :.***:*:::::.*** *:**:...    *:***:: : *:**:*..*******:* *

MG10127.1 	SSTSATPVDKQALYARSVMEPLFRDMENHLEKAKSGDAQAILQIHHVIMALGTLAHGFGD
NCU00134.1	SSTSTTPETDQALYARSVMEPLFSDMSVHLPRAKSGDAQAILQIHHIIMALGTLANGFAD
FG06659.1 	SSTQSTPADKQAMYARSVMDPLFTDMEGHLPRAKSGDAQATLQVHHIVMALGTLAHGFSD
AN8787.1  	CSIPTIPADKQVLYAQSVLSPVFVDMEKNLGSAKSGDARAVLQIHHDIMALGTLARGFSD
          	.*  : *  .*.:**:**:.*:* **. :*  ******:* **:** :*******.**.*

MG10127.1 	HAKPG--HQRQAPDKLVSAEFARAAEAILIALGQLNSRMDIRAACRSAFSKLLNVLGSAV
NCU00134.1	PNQSQNPNNQRTPPQAVSAEFSRASEAILVALNELNTNGEIRAACRSAFSRLLGVLGATI
FG06659.1 	WTPGITSNSHGPPDKAVSDEFSRAAEAILIALNQLNSSAEIRTACRSSFSKLLGVLGAAV
AN8787.1  	WQPGTSSPATQLPAPEVSEAFSQVSEATLVALESLKASFDIRTASRFAFSRLIGVLGSRI
          	     ..     *   **  *::.:** *:** .*::  :**:*.* :**:*:.***: :

MG10127.1 	LPQLPQWIEGLLSQSSSKDEMAMFLRLLDQVVYGFKTEISDVLNLLLTPLLQRVFGGLAE
NCU00134.1	LPQLPQWIEGLLSQSSSKDEMAFFLRLLEQIVYNFKGEIYNILDLLLTPLLQRVFAGLSE
FG06659.1 	LPQLPQWIEGLLSQSSSKDEMAMFLRLLDQVVFGFKSEIYEVLNMLLTPLLQRIFAGLTE
AN8787.1  	LPQLPRWIDGLLTQTSSRDEMALFLRLLDQVIFGFKGEIYNILDALLMPFLQRVFSGIAD
          	*****:**:***:*:**:****:*****:*:::.** ** ::*: ** *:***:*.*:::

MG10127.1 	PINGTDDEIQLGELRREYLTFLQIILDNDLGAVLVSET~NQGFFESIISSVVTLAKTGGH
NCU00134.1	PINGTDDEIQLQELRREYVSFVQVILINELGGVLVSTS~NQGVFESLVNSIMTIAKTIVH
FG06659.1 	PIAGTDDEIQLGELRREYLSFLQIILNNGLDGVLVSES~NQGFFEPMISSIIELAKT-LE
AN8787.1  	PTSGTDDEIHLAELKREYLNFLLAVLNNDLGAVIISER1NQPMFDTVITTIEHFAKD--A
          	*  ******:* **:***:.*:  :* * *..*::*   ** .*:.::.::  :**    

MG10127.1 	VNMVASRLAFCTLYRIVGVWGGPDVANISANPSAPTGTPTPAIPGFDQFMIERFHPVCFE
NCU00134.1	GNIVASRISFNVLARMAQQWGGPDVATIGENPTAN-GVPAPAFPGFDQFMLTQFHAACWE
FG06659.1 	GNVTSSRLAFTLMMRMASIWGGPDVATIAQNPTAPSGSPNPTFPGFEQFMIDRFHSTCWD
AN8787.1  	EDFTTAKMAFSVLSRMGSAWGGPDIAPAASN---GPSTSQVALPGFGQFMITRFSPLCWA
          	 :..:::::*  : *:   *****:*  . *    .. .  ::*** ***: :* . *: 

MG10127.1 	VLQDPQFNPSKDAQSKQVLNEIAALEQAIYVKTGNSFISHLQSSLFPALGIDG---TEFL
NCU00134.1	VMQDINFRPYADAQTRQILNEITGLEQIIYLKTGEKFISHCQTVTFPAVGMGA---EDFL
FG06659.1 	VIKNPNFRPFQDAQTKQVLTEIAGLEQIIYTKTGDVFIQELQNNLFPNLGVSG---DDFL
AN8787.1  	LPATPSFN-AKDAQAKQVLAEAGGLQRTIYSKMGMEYIEYLRDRELPGMGMGADLVEEYV
          	:    .*.   ***::*:* *  .*:: ** * *  :*.  :   :* :*:...   :::

MG10127.1 	RCMTTSTDKKTFGNYLQ~NLIKNRR2RVQEM
NCU00134.1	RALTSSTDRKAVMAYLQ~QLLKSRR~-----
FG06659.1 	RSLTTSTDKRQFASYLQ~NLLKSRQ~-----
AN8787.1  	GTLSR-LDLRGFRQFFP0QFIQRLS~A----
          	  ::   * : .  ::  ::::
```
